# Terpenoid-Induced Feeding Deterrence and Antennal Response of Honey Bees

**DOI:** 10.3390/insects11020083

**Published:** 2020-01-23

**Authors:** Nicholas R. Larson, Scott T. O’Neal, Ulrich R. Bernier, Jeffrey R. Bloomquist, Troy D. Anderson

**Affiliations:** 1 Department of Entomology, Virginia Tech, Blacksburg, VA 24061, USA; nicholas.larson@usda.gov; 2 Department of Entomology, University of Nebraska, Lincoln, NE 68583, USA; soneal3@unl.edu; 3 USDA Agricultural Research Service, Center for Medical, Agricultural and Veterinary Entomology, Gainesville, FL 32611, USA; Uli.Bernier@ARS.USDA.GOV; 4 Department of Entomology and Nematology, Emerging Pathogens Institute, University of Florida, Gainesville, FL 32611, USA; jbquist@epi.ufl.edu

**Keywords:** honey bee, terpenoids, behavior, feeding deterrence, olfaction

## Abstract

Multiple interacting stressors negatively affect the survival and productivity of managed honey bee colonies. Pesticides remain a primary concern for beekeepers, as even sublethal exposures can reduce bee immunocompetence, impair navigation, and reduce social communication. Pollinator protection focuses on pesticide application guidelines; however, a more active protection strategy is needed. One possible approach is the use of feeding deterrents that can be delivered as an additive during pesticide application. The goal of this study was to validate a laboratory assay designed to rapidly screen compounds for behavioral changes related to feeding or feeding deterrence. The results of this investigation demonstrated that the synthetic Nasonov pheromone and its terpenoid constituents citral, nerol, and geraniol could alter feeding behavior in a laboratory assay. Additionally, electroantennogram assays revealed that these terpenoids elicited some response in the antennae; however, only a synthetic Nasonov pheromone, citral, and geraniol elicited responses that differed significantly from control and vehicle detections.

## 1. Introduction

The value that insect pollination services provide to agriculture in both Europe and North America has been estimated at approximately $20 billion annually, while globally the estimate is around $212 billion annually [1]. It has also been estimated that 35% of the global human diet relies on crops that benefit from pollinators [2]. Global use of pesticides has almost doubled in the last 30 years [3], however, and honey bee (*Apis mellifera* L.) exposure to pesticides can result in both acute and sublethal effects that negatively impact colony health and survival [4,5,6,7]. For example, recent studies have reported that exposure to neonicotinoid insecticides can impair bee navigation, motivation to forage, social communication [8], increase their susceptibility to disease [9,10], and produce a wide range of other long-term detrimental effects [11,12], in addition to potential environmental and human health concerns that have been reported [13,14]. Given the importance of pesticides in modern agricultural systems and for ensuring food security now and in the future [15], there is an urgent need for novel approaches that mitigate the potentially harmful effects of agrochemicals on pollinating arthropods such as bees.

One possible approach that has been explored for decades is the incorporation of a repellent additive in pesticide mixtures in order to discourage bees from visiting blooming plants that have recently been treated with insecticides [16,17,18]. In practice, however, insect repellents have been developed primarily for personal protection to mitigate the spread of disease-causing pathogens by biting arthropods such as mosquitoes and ticks [19]. There have been a few studies demonstrating that 2-heptanone and isoamyl acetate, both alarm pheromones that are produced by bees, can act as repellents when applied to blooming plants [20,21]. Unfortunately, this line of research has yet to result in any commercial products or see widespread use for a variety of reasons, including the volatile nature of most spatial repellent compounds, which makes them challenging to employ in the field [22]. Rather than relying on a model based on spatial repellency, we propose that a more appropriate endpoint than repellency would be feeding deterrence. Consequently, the research findings described here investigate the use of a recently-developed protocol [23] for assessing behavioral changes in honey bees exposed to either a treated or untreated food source.

The initial goal of this work was to utilize *A. mellifera* as a model organism in order to validate a laboratory assay designed to rapidly screen compounds for behavioral changes related to feeding or feeding deterrence. Here, bee behavioral changes were characterized using a video-tracking protocol designed to monitor individuals within a Petri dish arena. Electroantennogram (EAG) assays were conducted to determine whether or not compounds that elicited behavioral changes were being detected via olfaction. As positive controls for avoidance or feeding deterrence, we employed isoamyl acetate and the commonly used repellent *N*,*N*-diethyl-meta-toluamide (DEET). Initially, the Nasonov pheromone was chosen for use as a negative control or attractant, as it is produced by workers and is used for orientation at the hive, recruitment when foraging, and during swarming [24]. The Nasonov pheromone consists of a mixture of terpenoids, including citral (*E* and *Z* conformations), geraniol, nerol, geranic acid, nerolic acid, and (*E*,*E*)-farnesol [24,25,26], though a synthetic Nasonov pheromone composed of citral, geraniol, and nerol is commonly used by beekeepers as a lure for swarm collection. Initial screens, however, revealed that the synthetic Nasonov pheromone elicited an avoidance response, or caused feeding deterrence, much like isoamyl acetate and DEET. To further investigate this effect, we tested the individual terpenoids found within the synthetic pheromone for behavioral changes within worker bees. Our findings revealed that these terpenoids alter honey bee behavior in a manner that is consistent with feeding deterrence.

## 2. Materials and Methods

### 2.1. Subjects

Honey bee colonies were maintained at the Price’s Fork Research Apiary of Virginia Tech (Blacksburg, VA, USA) using standard beekeeping practices. Worker bees were collected from frames located in the uppermost super of a colony to ensure that newly emerged and nurse bees were not collected prior to testing the compounds. The collected worker bees were maintained in ventilated plastic cages with approximately 50 bees per cage in an incubator at 32 °C with ca. 70% relative humidity. Bees housed in the lab overnight or longer are typically provided with ad libitum access to honey and a 50% solution of sucrose in water, but for this experiment the bees were provided only with water to avoid dehydration and were starved overnight prior to video-tracking experiments. For the EAG experiments, bees were collected from colonies maintained at the Pollinator Garden Apiary of the University of Nebraska (Lincoln, NE, USA) using standard beekeeping practices. The collected worker bees were taken from the uppermost super of the colony on the same day to avoid collecting newly emerged and nurse bees. Bees were temporarily housed in ventilated plastic cages prior to experimentation and anesthetized on ice to prepare the antennae for EAG recordings.

### 2.2. Compounds

**Figure 1:**
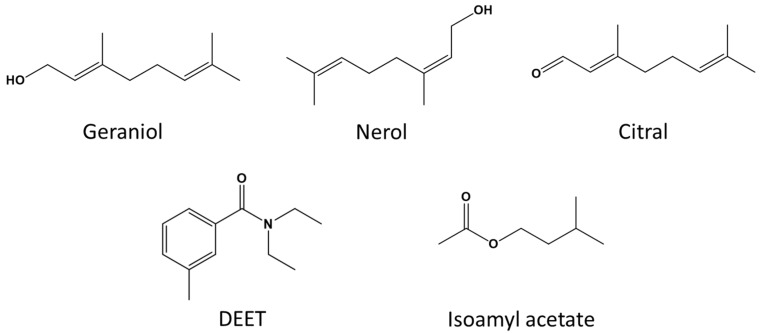
Chemical structures for the Nasonov terpenoids (geraniol, nerol, and citral), *N,N*-diethyl-meta-toluamide (DEET), and isoamyl acetate.

Synthetic Nasonov pheromone was purchased from Dadant & Sons Beekeeping Equipment (Chatham, VA, USA). Citral, geraniol, and nerol, in addition to isoamyl acetate and agarose, were purchased from Sigma-Aldrich Chemical Co. (St. Louis, MO, USA). OFF! Deep Woods^®^ Sportsmen (SC Johnson, Racine, WI, USA) was used as the source for DEET (98% active ingredient; *N,N*-diethyl-meta-toluamide; Figure 1).

### 2.3. Video-Tracking Recordings

A Basler acA-1300-60gm camera and EthoVision XT video recording software was used for behavioral recordings (Noldus Information Technology, Wageningen, the Netherlands). A light box was used to illuminate the assay arena with Cryon LED lights (Model HTP904E, Chatsworth, CA, USA) set to the red spectrum in order to avoid light bias of the bees. The light box and camera system were covered with a black plastic sheet to eliminate ambient light.

A previously described video-tracking protocol [23] was used to evaluate the effects of the synthetic Nasonov pheromone, citral, geraniol, nerol, isoamyl acetate, and DEET on the feeding behavior of bees. Briefly, sugar–agarose cubes were prepared by dissolving 8 g of sugar into 20 mL deionized water followed by the addition of 170 mg agarose. The sugar–agarose solution was heated and then poured into 1.5 cm × 1.5 cm × 0.3 cm molds. The pheromone, individual terpenoids, and DEET were added to the sugar–agarose solution, at the desired concentrations, prior to pouring the solution into the molds. The testing arena for an individual bee consisted of a single sugar–agarose cube that was placed into the center of a Petri dish. The space in each dish occupied by the sugar–agarose cube was designated the feeding zone. In order to ensure proper ventilation, holes were drilled into the lids of each Petri dish. A total of eight Petri dishes with untreated sugar–agarose cubes (control) and eight dishes with treated sugar–agarose cubes were positioned on top of a light box in a 4 × 4 grid pattern, then individual bees were placed into each dish. The dishes were arranged with treatments in alternating columns, for example, if the four dishes in the column on the left contained untreated sugar–agarose cubes, then the next column would have treated cubes, and so on. The video-tracking recordings were conducted with bees exposed to the sugar–agarose cubes for 10 min followed by another set of recordings with new bees exposed to the same cubes for another 10 min. Each video-tracking recording was conducted in duplicate. The time spent by the bees in the feeding zones was calculated using EthoVision video-tracking software (Noldus Information Technology, Wageningen, the Netherlands) and the data were statistically analyzed using a one-way ANOVA with a Tukey’s post-hoc test (GraphPad Prism 8.3.0, La Jolla, CA, USA) to identify significant differences between the means of each treatment group.

### 2.4. Electroantennogram (EAG) Recordings

EAG recordings were conducted as previously described [27]. Chemicals were diluted into either a hexane or ethanol (Sigma-Aldrich, St. Louis, MO, USA) carrier solvent at 10 µg/µL (1% *w*/*v*) and stored at 4 °C prior to the EAG recordings. For the concentration response bioassays, a serial dilution of each chemical was prepared using either a hexane or ethanol carrier solvent. A 10 µL aliquot of each chemical was applied to a strip of Whatman No. 1 filter paper (Sigma-Aldrich, St. Louis, MO, USA) and then briefly allowed to dry before inserting the chemical-treated paper into a Pasteur pipette (15 cm long) for the EAG recordings. EAG recordings were conducted using Ag–AgCl electrodes inserted into glass capillary tubes filled with electrogel (Spectro 360, Parker Laboratory, NJ, USA). Thin-walled glass capillary tubes (World Precision Instruments, Sarasota, FL, USA) were pulled to a fine tip with a P-97 Flaming/Brown Micropipette puller (Sutter Instrument, Novato, CA, USA) using the following settings: heat = 500; pull = 25; velocity = 200; time = 500; and pressure = 500. Bee antennae were dissected below the scape with micro scissors under a dissection microscope. The glass capillary tube was cut at the tip with micro forceps to fit the base of a bee antenna, while the other end of the capillary tube was attached to a reference electrode. The reference electrode was then positioned so that the tip of the excised antenna was near the recording electrode. A second glass capillary tube was prepared as described above to fit the tip of the bee antenna, and the other end of the capillary tube was attached to a recording electrode. The recording electrode was connected to a high-impedance direct-coupled (DC) amplifier (Ockenfels Syntech Gmbh, Kirchzarten, Germany) and change in baseline DC voltage (mV) was analyzed using EAG Pro 1.1 software (Syntech, Stilwell, KS, USA).

A bee antenna was exposed to a compound by inserting a Pasteur pipette containing a chemical-treated filter paper into the opening of a glass mixing tube that was positioned perpendicular to the suspended bee antenna. This mixing tube provided a constant flow of humidified air over the prepared bee antenna. A 1 s puff of air was passed through the Pasteur pipette and into the mixing tube to deliver the chemical to the bee antenna. An empty Pasteur pipette and hexane or ethanol treated filter paper served as a control and carrier solvent treatment, respectively. A total of two to seven bee antennae were exposed to the compounds in a randomized order. The antennae were allowed to return to the baseline voltage before applying each compound. For the concentration response bioassays, a new antenna was used for each concentration of compound (*n ≥* 3). The change in baseline voltage was recorded at 10 min intervals. A baseline voltage of the bee antennae was generated prior to compound exposure. A lowest detection point was determined by the program, indicating the voltage at the lowest point after a stimulus was applied. The absolute values of these two points for each stimulus were calculated and the baseline value was subtracted from the lowest detection point to give the change in baseline voltage (mV). The change in baseline voltage was statistically analyzed using an ANOVA with a Tukey’s multiple comparison test (GraphPad Prism 8.3.0, La Jolla, CA, USA).

## 3. Results

### 3.1. Video-Tracking Recordings

**Figure 2:**
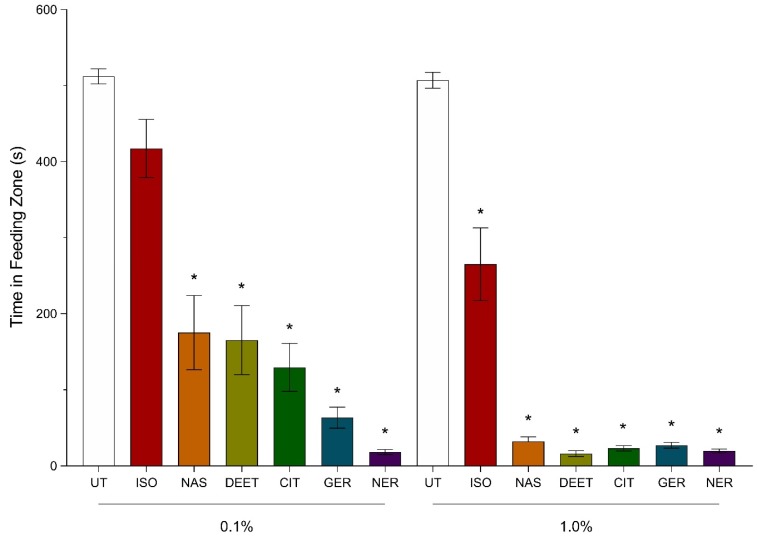
Efficacy of Nasonov terpenoids and *N,N*-diethyl-meta-toluamide (DEET) at 0.1 and 1.0% (*v*/*v*). Untreated (UT), isoamyl acetate (ISO), synthetic Nasonov pheromone (NAS), citral (CIT), geraniol, (GER), and nerol (NER). A video tracking protocol with individual bees in Petri dishes was used to observe behavior changes when exposed to sugar–agarose cubes treated with the terpenoids. The UT controls were sugar–agarose cubes with no terpenoid or DEET treatment. The DEET treated sugar–agarose cubes were screened as a positive control. Vertical bars represent mean ± standard error. Asterisks above the bars indicate a significant difference between the mean of the terpenoid- or DEET-treated bees compared to the UT bees using a one-way ANOVA with a Tukey’s post-hoc test (*p* < 0.0001).

The amount of time spent by bees in the feeding zones containing sugar–agarose cubes treated with Nasonov terpenoids and DEET at 0.1% (*v*/*v*) was observed as untreated (more time spent) = isoamyl acetate > synthetic Nasonov pheromone = DEET = citral > geraniol > nerol (less time spent). Bees were recorded in the feeding zone of control sugar–agarose cubes for 512 ± 10 s whereas bees exposed to the DEET treated sugar–agarose cubes were in the feeding zone for 166 ± 45 s. Nerol reduced the time spent by bees in the feeding zone greater than that spent by bees in the control, isoamyl acetate, synthetic Nasonov pheromone, and DEET treatment. Bees exposed to nerol-treated cubes at 0.1% (*v*/*v*) spent 18 ± 3 s in the feeding zone (Figure 2). This reduction was not significantly different from that observed with geraniol and citral. Geraniol and citral reduced the time spent by bees; however, the reduction in time was not significantly different from that of synthetic Nasonov pheromone and DEET. Bees exposed to 0.1% (*v*/*v*) synthetic Nasonov pheromone, DEET, geraniol, and citral spent 175 ± 49 s, 166 ± 45 s, 64 ± 14 s, and 130 ± 32 s, respectively. Bees exposed to isoamyl acetate at 0.1% (*v*/*v*) spent 417 ± 38 s in the feeding zone, which was not significantly different from the control treatment (Figure 2).

The amount of time spent by bees in the feeding zone containing sugar–agarose cubes treated with Nasonov terpenoids and DEET at 1% (*v*/*v*) was observed as untreated (more time spent) > isoamyl acetate > synthetic Nasonov pheromone = geraniol = citral = nerol = DEET (less time spent). Bees were recorded in the feeding zone of the untreated sugar agarose cubes (control treatment) for 507 ± 10 s, whereas bees exposed to DEET-treated sugar–agarose cubes were in the feeding zone for 16 ± 4 s. Nasonov terpenoids reduced the amount of time spent in the feeding zone by 94% compared to the control treatment. Bees exposed to synthetic Nasonov pheromone-, geraniol-, citral-, and nerol-treated sugar–agarose cubes spent 32 ± 6 s, 27 ± 4 s, 23 ± 3, and 19 ± 2 s, respectively, and were not significantly different from the feeding time spent by bees exposed to DEET-treated sugar–agarose cubes. Bees exposed to isoamyl acetate-treated sugar–agarose cubes spent 265 ± 48 s in the feeding zone, which was significantly different than the time spent by bees in the feeding zones of the control treatment (Figure 2).

The amount of time spent by bees in the feeding zone when exposed to 0.1% (*v*/*v*) synthetic Nasonov pheromone, citral, isoamyl acetate, and DEET was significantly different from the time spent in the feeding zone when bees were exposed to 1% (*v*/*v*) of those compounds. The reduction in time when increasing the concentration of synthetic Nasonov pheromone, citral, isoamyl acetate, and DEET by 10-fold was 89%, 88%, 36%, and 90%, respectively (Figure 2). Increasing geraniol and nerol concentrations by 10-fold did not significantly reduce the amount of time spent by bees exposed to the treated sugar–agarose cubes (Figure 2).

### 3.2. Electroantennogram (EAG) Recordings

**Figure 3:**
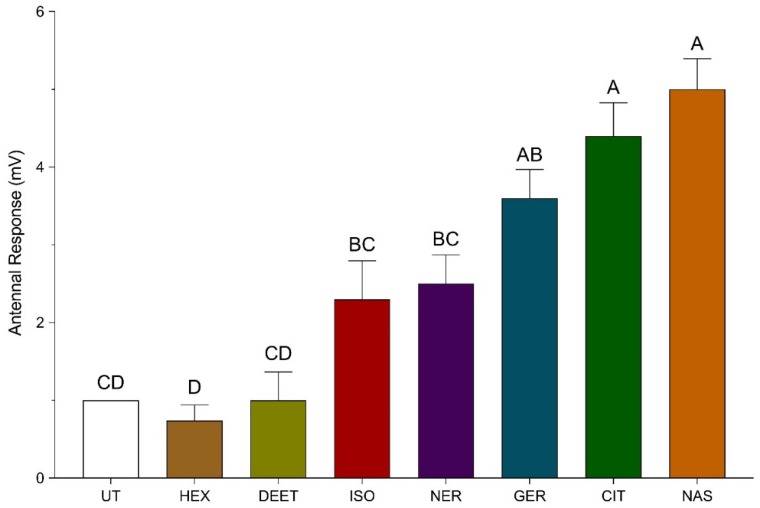
Electroantennogram (EAG) recordings of bee antennae exposed to synthetic Nasonov pheromone (NAS), citral (CIT), geraniol (GER), nerol (NER), *N,N*-diethyl-meta-toluamide (DEET), and isoamyl acetate (ISO). The change in baseline voltage (mV) was calculated by subtracting the absolute value of the baseline voltage from the absolute value of the response voltage. An empty Pasteur pipette and hexane treated filter paper served as a control (UT) and carrier solvent treatment (HEX), respectively. Vertical bars represent the mean ± standard error. Different letters above the bars indicate a significant difference between treatments using a one-way ANOVA with a Tukey’s multiple comparison test (*p* < 0.0001).

Bee antennae were stimulated with pheromones to examine their olfactory response to these compounds using EAG recordings. The change in baseline voltage for bee antennae exposed to the pheromones were observed as isoamyl acetate (low activity) = nerol = geraniol < citral = synthetic Nasonov pheromone (high activity; Figure 3). A control (air only) and carrier solvent (hexane or ethanol only) treatment produced a 0–2 mV change in baseline voltage of the bee antennae. The change in baseline voltages for bee antennae exposed synthetic Nasonov pheromone, citral, geraniol, nerol, and isoamyl acetate were 5.0 ± 0.4, 4.4 ± 0.4, 3.6 ± 0.4, 2.5 ± 0.4 mV, and 2.5 ± 0.5 mV, respectively, which were significantly elevated relative to the controls. There was no significant difference in the responses of bee antennae exposed to synthetic Nasonov pheromone, citral, and geraniol; however, the change in baseline voltage of bee antennae exposed to synthetic Nasonov pheromone and citral was significantly higher than that of antennae exposed to nerol and isoamyl acetate. The change in baseline voltage of bee antennae exposed to nerol and isoamyl acetate was not significantly different than that provided by the control and solvent treatments. Furthermore, the change in baseline voltages for bee antennae exposed to citral, geraniol, and nerol was not significantly different from one another, which suggests a similar olfactory response to the Nasonov terpenoids.

## 4. Discussion

While pesticide exposure threatens the health and survival of managed and wild bees alike, continued pesticide use is required to meet the dietary needs of the world’s population [4]. It has been suggested that repellents can mitigate the interaction time between foraging bees and a pesticide-treated crop; however, there has been little success in identifying and developing commercial bee repellant additives [22]. Here, we focused on developing a screening method to assess compounds for the induction of avoidance behavior or feeding deterrence in bees, rather than repellency in the classical sense. Our findings demonstrate that terpenoids found in honey bee Nasonov pheromone can alter the behavior of a worker bee in the presence of a treated food source as effectively as DEET at similar concentrations of 1% (*v*/*v*), and additionally elicit bee antennal responses, suggesting that bees may be responding to olfactory cues.

The Nasonov pheromone is used by bees as an orientation pheromone and the synthetic Nasonov pheromone is used by beekeepers as a bee attractant that is reportedly five times more attractive to swarms than other attractants [28]. Our laboratory findings appear to contradict this observation, though this may be partially explained by the small size of the Petri dish used for the observational recordings. Honey bees prefer nest sites, including swarm boxes, that are approximately 31 L in volume [29]. This is significantly larger than the volume of the Petri dishes (0.095 L) used for the observational recordings of this study. The smaller volume allows for the concentration of the compound to be much higher than what would typically be found in a swarm box or natural nest site. Additionally, swarm boxes and natural nest sites have higher airflow compared to that present in a Petri dish, allowing for more rapid dispersion of the pheromone into air. The disparity in observed behaviors may also be explained by the use of synthetic Nasonov pheromone. Natural Nasonov pheromone is a mixture of six monoterpenes: (*E*) citral, (*Z*) citral, geraniol, nerol, geranic acid, nerolic acid, plus the sesquiterpene (*E*,*E*)-farnesol, with the approximate ratio of 1:1:1:100:75:12.5:50. The synthetic Nasonov pheromone used in this study consists of a 1:1:1 mixture of citral, geraniol, and nerol [24,25,26,30]. The most important components for clustering are reported to be (*E*) citral, geraniol, and nerolic acid [31], though other studies have suggested that all seven terpenoids are required to achieve a full attractive effect [32]. Numerous studies have demonstrated that insect behaviors are strongly associated with naturally occurring blends of individual compounds, suggesting that the blend is more important than the individual components [33].

Terpenoids have been routinely studied for their potential use as insecticides, repellents, and antifeedants [34,35]. Geraniol has been shown to reduce feeding in the freshwater snail, *Planobis corneus* L., and has repellent activity to the parasitic mite, *Varroa jacobsoni* (Oudemans) [36,37]. Geraniol has been found to repel mosquitoes and sand flies when added to candles, has been demonstrated to protect against several mosquito species when used as the active ingredient in a repellent lotion [38,39], and has been reported to provide significant protection to caged mice from *Aedes aegypti* mosquitoes [40]. However, geraniol also has been reported to attract forager bees to feeder stations positioned in a large lawn [32], again suggesting differences based on the volume of airspace. How geraniol application would affect the attractiveness of a blooming plant to bees remains to be seen, as this potentially differs from the incorporation of geraniol into an artificial food source in a feeder that they have been trained to locate.

Citral has been found to act as an antifeedant, similar to geraniol, in *P. corneus* L. [37]. Citral has been identified in the mandibular glands of two ant species, *Atta sexdens rubropilosa* (Forel) and *Ancanthomyops claviger* (Roger), where it acts as an alarm pheromone and a defensive chemical, respectively [41]. Citral is found in Nasonov pheromone in two different conformations (*E*) citral and (*Z*) citral [26,42]. The citral used within this study was a mixture of both isomers 1:1. It has been reported that (*E*) citral is more attractive than (*Z*) citral, and there is an enzymatic reaction within the Nasonov gland that converts geraniol into (*E*) citral before the Nasonov pheromone is released by bees [30,32]. It is possible that this change in concentration of the citral isomers is important for attractancy. This study found citral to elicit avoidance from the treated food source in individual bees, which may indicate the citral isomer ratios are different from those found in the naturally occurring Nasonov pheromone.

Nerol is the geometric isomer of geraniol and has been shown to diminish the attractiveness of a synthetic Nasonov pheromone blend containing all seven terpenoids when compared to a similar blend without nerol [30]. The present study found nerol to be the most potent elicitor of the avoidance behavior. While both geraniol and citral diminished in their efficacy when diluted 10-fold, the efficacy of nerol was preserved. Nerol has been found to be an alarm pheromone in the stingless bee *Trigona fulviventris* (Guerin) [43]. It has been found to be as effective as DEET in repelling the tick *Rhipicelphus appendiculatus* (Neumann) and the red flour beetle *Triboleum castaneum* (Herbst) [44,45]. Nerol has also been shown to attract the dwarf honey bee, *Apis florea* (Fabricius)*,* to treated feeding dishes, but as concentrations increase, attraction diminishes resulting in an increase in the average number of bees visiting untreated feeding dishes [46]. Our results demonstrated that nerol altered behavior at a concentration 160-fold lower than the concentration reported by Naik et al. [46], most likely due to the differences in experimental approach, as the dwarf honey bees were exposed to nerol in a larger arena than the Petri dishes used for this study.

Isoamyl acetate is an active component of the alarm pheromone used by honey bees to recruit defenders to an imposing threat [47]. Here, it was observed to reduce the amount of time that a bee will spend in the feeding zone in this study. Previous studies report that isoamyl acetate repels honey bees and bumble bees from crops treated with it [20]. Boch et al. report isoamyl acetate to have repellent activity at concentrations above 200 µg/µL [48], compared to 9 µg/µL used in this study. Bee attraction to isoamyl acetate may require additional visual input to elicit the desired behavior. For example, multiple studies reported that isoamyl acetate alone was not enough to trigger a defensive behavior, but also required additional input such as movement of the threat to the bee or colony [49,50,51]. Consequently, the behavioral changes observed with exposure to isoamyl acetate are likely very context-dependent.

This study found that the synthetic Nasonov pheromone, citral, and geraniol elicited the strongest EAG responses compared to those of untreated bees. The average change in baseline voltage for synthetic Nasonov pheromone was slightly higher than citral whereas the responses to geraniol were slightly less than citral. Similar results have previously been reported, in which the EAG responses of honey bee antennae to Nasonov were greater than those produced by geraniol and nerol [52]. Single sensillum recordings of the olfactory system of honey bees show greater sensitivity to citral compared to geraniol [53,54]. Honey bees are reported to have more olfactory receptor neurons for citral than geraniol, and this response may be associated with the number of receptor neurons that are attuned specifically to each of the compounds [53,54]. EAG records the sum of receptor potential, thus having more receptors selective for a specific compound would result in a larger response. Additionally, this might explain the higher voltage change for synthetic Nasonov pheromone relative to the other components, since there are more olfactory receptor neurons activated by the blend of citral, geraniol, and nerol. Nerol elicited the lowest EAG response of the Nasonov terpenoids, which may be due to it being a *cis-*isomer. A previous study found that *cis*-isomers such as nerol were significantly less effective than *trans-*isomers at inducing EAG responses in the hymenopteran *Sirex noctilio* (Fabricius) [55]. In one study, the bumble bees *Bombus hypnorum* L. and *Bombus terrestris* L. were reported to be more sensitive to geraniol than nerol [56], while another found that isoamyl acetate elicited a stronger EAG response in honey bee antennae compared to synthetic Nasonov pheromone and the terpenoids [57].

Honey bees mark threats to their hive with isoamyl acetate to elicit recruitment of additional defenders to respond to the threat. This in turn, results in a positive feedback loop for increased pheromone release and continued recruitment [47,51]. Masson and Arnold (1984) reported stronger EAG responses to 100% isoamyl acetate, relative to the terpenoids comprising the Nasonov pheromone [57]. The use of pure compounds by Masson and Arnold, when compared to our findings using 100-fold dilutions of the compounds, might explain why isoamyl acetate elicited EAG responses below the synthetic Nasonov pheromone and its individual terpenoids. Additionally, from a practical standpoint, a bee pheromone that excites a large portion of the colony might require a concentration threshold to be triggered before the occurrence of a biological response. While isoamyl acetate did not elicit a significant EAG response of the bee antenna in this study, it might be related to the low number of antennae used for the EAG recordings. Therefore, an increase in the number of antennae exposed to isoamyl acetate and nerol might decrease the variable responses detected with the EAG recordings.

## 5. Conclusions

The development of laboratory assays to screen model organisms for behavioral changes is essential for expediting novel research objectives in a cost-effective manner. In our effort to validate a laboratory behavior assay with a known attractant we discovered that, in this assay, the typically attractive synthetic Nasonov pheromone elicits avoidance or feeding deterrence within individual worker bees. This study’s efforts were subsequently redirected to characterize these behavioral changes by investigating several naturally occurring terpenoids that are constituents of the natural honey bee Nasonov pheromone. The evidence presented here demonstrated that these terpenoids were as effective as DEET in eliciting avoidance behavior to a treated food source by individual honey bees within a Petri dish arena. Further studies are needed to expand the area of exposure and to determine the retention time of the avoidance effects, but more importantly to investigate how likely these compounds are to inhibit feeding in a field setting. The evidence provided here should be used within the application of structure–activity relationships to drive the screening process of candidate chemistries, leading to the possible discovery of a highly effective feeding deterrent that could ultimately be used in conjunction with applied pesticide formulations for the protection of pollinators.
